# Novel occupational therapy interventions may improve quality of life in older adults with dementia

**DOI:** 10.1186/1755-7682-7-26

**Published:** 2014-05-20

**Authors:** Prakash Kumar, Sarvada Chandra Tiwari, Ashish Goel, Vishnubhatla Sreenivas, Nand Kumar, Rakesh Kumar Tripathi, Vineet Gupta, Aparajit Ballave Dey

**Affiliations:** 1 Department of Geriatric Medicine, All India Institute of Medical Sciences, New Delhi 110029, India; 2 Department of Geriatric Mental Health, King George’s Medical University, Lucknow, India; 3 Department of Medicine University College of Medical Sciences, Delhi, India; 4 Department of Biostatistics, All India Institute of Medical Sciences, New Delhi, India; 5 Department of Psychiatry, All India Institute of Medical Sciences, New Delhi, India; 6 University of Pittsburgh Medical Center (UPMC) Mercy, Pittsburgh PA 15219, USA

**Keywords:** Dementia, Occupational therapy, Quality of life, WHOQOL-BREFF

## Abstract

**Background:**

Dementia is a major health problem in advancing age with no definitive treatment. Occupational therapy interventions are recognized strategies in treatment of dementia. Quality of life (QOL) assessment has been reliably used as an objective index of an individual’s well being pertaining to interventions in dementia. A randomized controlled trial was conducted to study the effects of a novel occupational therapy program in improving QOL of subjects having mild to moderate dementia.

**Methodology:**

273 subjects older than 60 years were screened. 196 were excluded having cognitive impairment with no dementia (CIND). Remaining 77 subjects after satisfying DSM IV criteria for diagnosis of dementia were included in the study and were randomly assigned to experimental and control groups. Experimental group received a novel occupational therapy regimen along with medical treatment, while control group received only medical treatment for 5 weeks. Outcome measures included standard occupational therapy assessment and WHOQOL-BREF. Subjects were assessed at baseline and post intervention.

**Result:**

The mean age of participants was 69.39 years with male preponderance (80.5% male, 19.5% female). The quality of life (QOL) scores of physical and psychological domain in experimental groups significantly increased from 37.30 ± 5.42 and 45.13 ± 3.52 to 45.43 ± 7.32 and 51.50 ± 6.46 respectively. The QOL scores in social and environmental domains did not change significantly. The QOL scores in control groups declined in all domains with statistical significance found in social and environmental domain. (29.67 ± 4.58 and 38.49 ± 1.77 to 28.45 ± 5.26 and 38.18 ± 2.15 respectively).

**Conclusion:**

This novel occupational therapy program improved the short term physical performance and psychological well being domain of quality of life in older adults with dementia. An improved physical performance is achieved by physical exercise of novel program and it creates sense of independency, increased motivation, positive outlook and reduced behavioral and psychological symptoms. The long term effects of the intervention can be ascertained in a study with longer period of intervention and follow-up.

**Trial registration:**

[CTRI/2014/01/004290]

## Introduction

Dementia is a common health problem in old age. It involves progressive cognitive decline beyond normal aging process and leads to progressive deterioration of physical and social functioning. Most patients with dementia lose autonomy and independence; and require continuous care and support [1]. Dementia affects almost every eighth person over the age of 65 years and every third person over the age of 85 years [1]. Dementia is caused by several pathological states and there is no definite medical management to reverse or arrest the disease process. This necessitates exploration of non-pharmacological management strategies, to improve functional status and quality of life (QOL) of patients and reduce need for care [2].

Alzheimer’s disease (AD) and vascular dementia (VaD) account for the majority of dementias. In most clinical descriptions AD is considered as the prototype of the syndrome. The clinical manifestations of dementia progress through various stages depending on the involvement of critical areas of the brain [3]. Clinical staging of dementia is based on assessment by established tools and is indicative of deficits in functioning in activities of daily living (ADL), speech, various other parameters of cognitive function and degree of dependence on care giver. Clinical staging often guides management strategies. Management of dementia remains unsatisfactory. Choline esterase inhibitors and NMDA receptor antagonists are used for cognitive symptoms along with various anti-psychotic drugs for behavioral symptoms. Various non-pharmacological strategies for cognitive symptoms have also been developed for management of dementia. A systematic review has revealed the effectiveness of non-pharmacological interventions similar to the currently available drug treatment, but without any side effects. The primary focus of occupational therapy is to improve patients’ ability to perform activities of daily living, promote independence, reduce caregiver burden and ultimately improve quality of life. In this study the utility of occupational therapy interventions has been assessed in the setting of cognitive decline and dementia.

QOL is a subjective assessment of several dimensions to evaluate both positive and negative aspects of life [4]. QOL in the context of physical and mental health is referred to as health related quality of life (HRQOL). HRQOL instruments can be generic or disease specific. The World Health Organization Quality of Life Assessment (WHOQOL), a generic scale, has been translated to several Indian languages and is widely used by researchers in India while assessing impact of interventions in various disease conditions. QOL for patients with dementia is of importance to patients and in addition, to their family members and care providers. However, QOL in dementia is a difficult concept in view of highly subjective and perceptive nature of assessment by definition [4]. Consequently there is no consensus on definition of QOL in dementia [5], though there is a general agreement that assessment of quality of life of patients with dementia should spread over several domains such as mood, affect, participation in activities, enjoyment of these activities, socializing, exercising, reading, doing crafts , appetite, sleep, and disturbances in behavior etc [5]. Notwithstanding these challenges, QOL assessment needs to be incorporated into the measurement of benefits of care as the central theme of clinical intervention and research [6]. According to the International Working Group on Harmonization of Dementia drug Guidelines, cognitive function and the ability to perform activities of daily living in addition to mood and affect needed to be included in QOL assessment in dementia [7]. Incorporating all these concepts and opinions, a disease specific QOL assessment strategy for dementia is available as DEMQOL, which is however not yet translated in Indian languages [8].

Pharmacological management of AD, VaD and other dementias is mostly symptomatic with no impact on disease progression. As a result several non-pharmacological strategies for treatment have been considered to complement with standard drug therapy in order to improve QOL. In one such strategy, concepts and interventions of occupational therapy have been included [9]. Occupational therapy provides interventions related to awareness of the occupational identity, self-care , productivity and leisure activities [9]. Previous studies have reported relationship between cognition on occupational participation and performance [10,11]. Utilizing these concepts, dementia patients can be supported with occupational therapy to maintain an optimal level of performance in daily lives [12]. In addition, care givers in the family or in institution can adopt occupation therapy techniques to stimulate patient performing daily activities, prevent disruptive behavior, wandering, and aggression. These interventions can also reduce caregiver stress and burden. In view of cognitive disability of these patients, the method of assessment needs to be based on two patient-centric models: the model of human occupation [13] and the Canadian model of occupational performance [14]. In turn, the interventions for these models should be based on biomechanical [15] and cognitive disability framework of reference [16]. The present study was designed to evaluate the impact of a novel occupational therapy program on QOL of patients with mild to moderate dementia by employing the Hindi version of WHOQOL-BREF questionnaire.

## Methodology

### Study design, setting and period

This open label randomized control trial was carried out at the Memory Clinic of the Department of Geriatric Medicine, All India Institute of Medical Sciences (AIIMS), New Delhi and Department of Geriatric Mental Health, King George Medical University (KGMU), Lucknow, India between November 2010 and May 2013. An experimental pre and post-test control group design was chosen for the study.

The protocol was reviewed and approved by the Ethics Committees of both institutions. A written Informed consent was taken from subjects or their primary caregivers prior to Inclusion in the study.

### Randomization

Concealment technique was used for randomization by a biostatistician who was not involved in conducting the study. Computer generated table was formed to allocate the subjects to either the experimental occupational therapy or the control group. In this open label randomized control trial the groups were classified by the level of dementia (mild or moderate).

### Recruitment of cases

**Figure 1:**
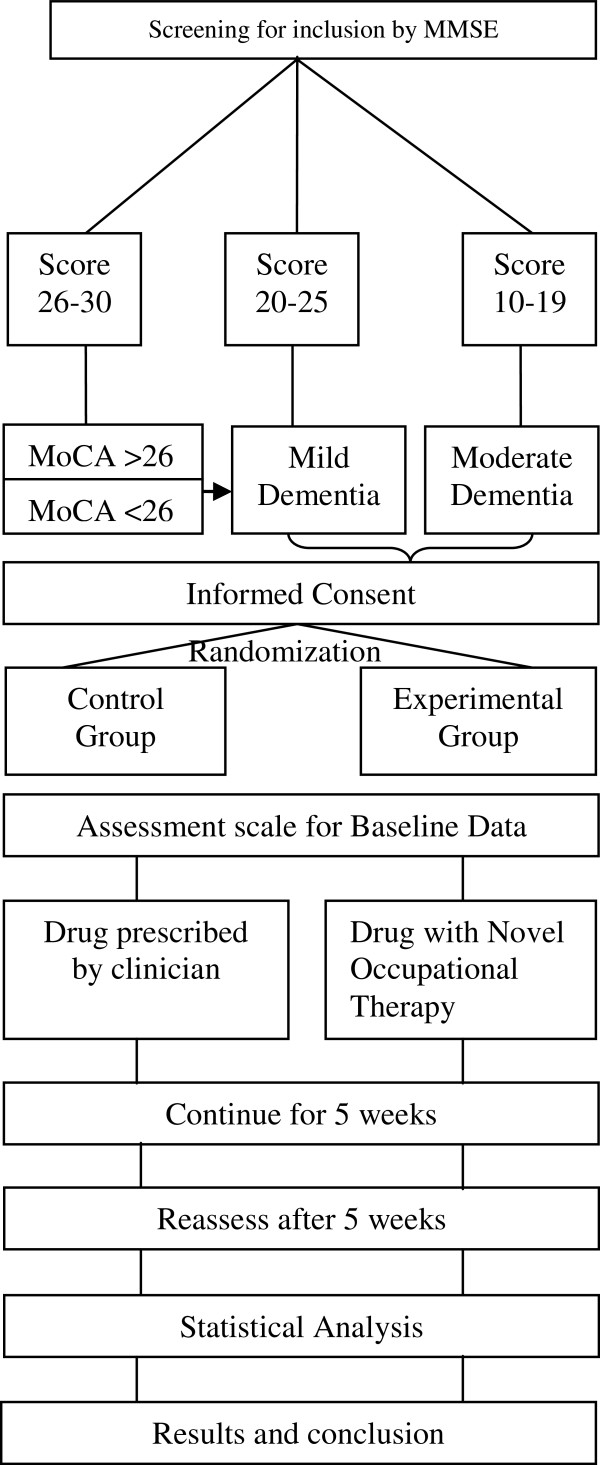
Study Flow (Schematic View).

Patients attending the Memory Clinic at AIIMS and KGMU were screened for dementia. The diagnosis of dementia was established by a geriatrician or a psychiatrist based on DSM IV criteria, screening for cognitive impairment [scores ≤ 23 on the Mini Mental State Examination (MMSE) scale [17] and confirmation by detailed neurological evaluation, assessment of activities of daily living and neuropsychological testing using the Clinical Dementia Rating Scale and the Blessed Dementia Rating Scale as has been reported earlier [18].Patients with MMSE score between 11 and 23; at least five years of formal schooling; and ability to read and understand simple sentences were recruited for intervention. Patients with severe dementia (MMSE < 11), severe depression, severe behavioral or psychological symptoms, or requiring nursing care for severe medical illness as were excluded from occupational therapy program (OTP) intervention. The study flow is shown in Figure 1.

### Study intervention

The OTP was developed through a process of consultation and following a pilot study in the Memory Clinic at AIIMS. The pilot study provided information on (a) feasibility, (b) sampling (c) design and (d) the OTP interventions to be used [19]. The details of the novel OTP have been published previously [20,21].

The intervention, including a total of 10 treatment session of 70 minutes each, was implemented for 5 weeks. Each session contained the following interventions:

1. Relaxation for 10 minutes involved alternately tensing and releasing different group of muscles, in order to gain awareness of the difference between a tense and a relaxed state [22].

2. Physical exercises for 10 minutes aimed at maintaining strength, mobility, circulation and general health which included range of movement exercise, medicinal ball training, imitating movement of another person and squatting at different levels [23].

3. Personal activities for 15 minutes which included a) personal care of body involving care of hair, skin, nails and teeth; general cleanliness, personal enhancement, dressing and undressing of upper and lower garments; b) household tasks such as arranging bed sheets, gardening and counting currency.

4. Cognitive exercise for 20 minutes [24] included loud reading, dual task activity (reading while walking, writing or drawing while listening music), solving picture puzzle, clay color activity, and neurobic exercise [25] (e.g. brushing teeth with the non-dominant hand).

5. Recreational activity for 10 minutes [26] included domestic duties, work, travel or specific treatment. These were balanced with recreational activity such as viewing television, playing indoor games, table games, quizzes, carom, Chinese checkers, telling story, singing, and participating in organized social events.

The experimental group received the novel OTP along with the standard medical treatment, and control group received the standard medical treatment for a period of 5 weeks similar in all respects to the study group except the therapeutic program under guidance of a trained occupational therapist (author PK).

### Measurement of outcome

Cases and controls were assessed at baseline with Hindi version of WHOQOL-BREF [27] for quality of life along with standard clinical and general occupational therapy assessment instrument. The WHOQOL-BREF consists of 26 items. Each item uses a Likert-type five-point scale. These items were grouped under four domains vis-à-vis (a) physical health and level of independence (seven items assessing areas such as presence of pain and discomfort; dependence on substances or treatments; energy and fatigue; mobility; sleep and rest; activities of daily living; perceived working capacity); (b) psychological well being (eight items assessing areas such as affect, both positive and negative self concept, higher cognitive functions; body image and spirituality), (c) social relationships (three items assessing areas such as social contacts, family support and ability to look after family; sexual activity) and (d) environment (eight items assessing areas such as freedom; quality of home environment; physical safety and security and financial status; involvement in recreational activity; health and social care: quality and accessibility). There were two items that were examined separately: one asking about the individual’s overall perception of QOL and the other asking about the individual’s overall perception of his or her health. WHOQOL-BREF domain scores are scaled in a positive direction (higher scores denote higher quality of life). The raw score of each domain was calculated and transferred into range between 0-100 for compatibility with the scores used in WHOQOL-100 using the standard guidelines [28].

### Data analysis

The data was analyzed using the SPSS software (version 13, SPSS inc, USA). Level of significance was taken at p < 0.05. Two-sample *t*-test was used for analysis between groups and paired sample *t*-test for within groups for continuous data. Chi square was used for comparing categorical variables.

## Results

273 older subjects were screened for the study. 196 were excluded and remaining 77 patients were included after satisfying DSM IV criteria for diagnosis of dementia and inclusion criteria mentioned above. They were randomly assigned to experimental and control groups. The mean age of subjects in control group was 69.85 years and 69.42 years in experimental group with 80.5% male and 19.5% female. The range was 60-83 and 60-81 years in control and experimental groups respectively. The experimental and the control groups were comparable at baseline and the demographic details are shown in Table 1. Differences in gender (p = 0.559), handedness (p = 0.742), occupation (p = 0.220), education (p = 0.752), marital status (p = 0.470), living arrangement (p = 0.330), and cognitive impairment (p = 0.105) between different groups were not statistically significant.

**Table 1:** Baseline comparison between control and experimental group

		**Control group (%)**	**Experimental group (%)**	**p-Value**
**Gender**	Male	32(78.0)	30 (83.3)	0.218
	Female	9 (22.0)	6 (16.7)	
**Handedness**	Right	35 (85.4)	32 (88.9)	0.724
	Left	6(14.6)	4(11.1)	
**Occupation**	Unemployed	12(29.2)	4(13.3)	0.113
	Employed	1(2.4)	4(13.3)	
	Retired	18(43.9)	13(43.3)	
	Business	7(17.0)	3(10.0)	
	Farming	3(7.3)	6(20.0)	
**Education**	Primary	15(36.5)	9(30.0)	0.455
	Class 12	11(26.8)	12(40.0)	
	Graduate	10(24.3)	8(26.7)	
	Postgraduate	5(12.2)	1(3.3)	
**Marital status**	Single	2(4.9)	1(3.3)	0.456
	Married	34(82.9)	22(73.3)	
	Widow/widower	5(12.2)	7(23.3)	
**Living arrangement**	Living alone	2(4.9)	1(3.3)	0.542
	With spouse	22(53.7)	12(40.0)	
	Family with spouse	17(41.5)	17(56.7)	
**Dementia**	Mild	32(78.0)	22(61.1)	0.105
	Moderate	9(22)	14(38.9)	

**Figure 2:**
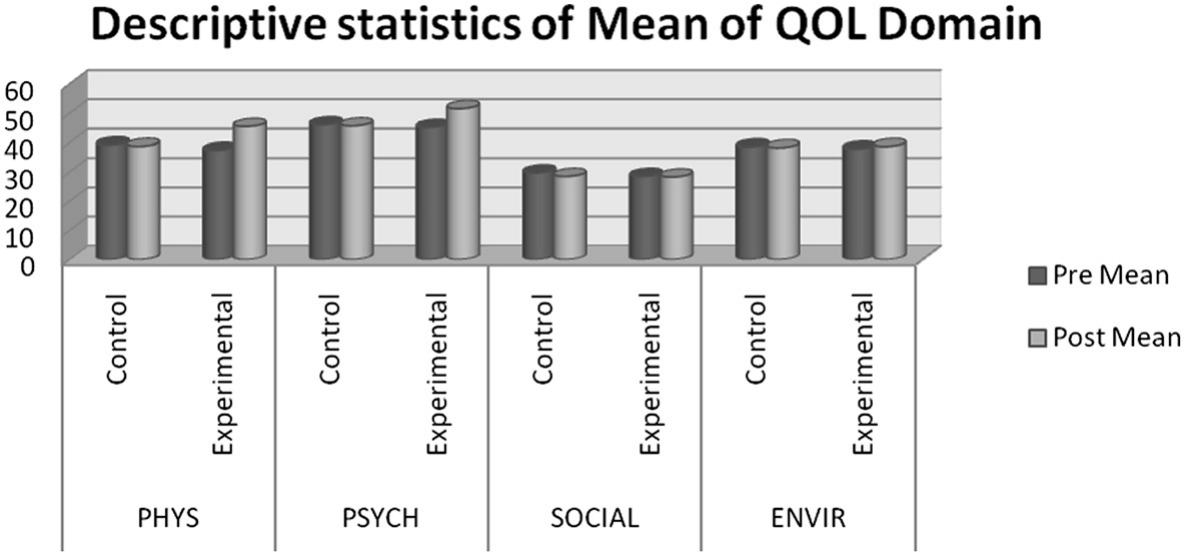
Showing mean value of pre-post WHO-QOL domain.

QOL assessments done before and after the intervention in the control group and the intervention group have been presented in Table 2. The overall quality of life (WHOQOL-BREF) improved significantly in the experimental group (p < 0.001) showing the effectiveness of program; whereas in the control group it significantly declined (p = 0.011) as shown in Figure 2. While the mean value of physical and environmental domain in control group declined (p = 0.057, 0.044) respectively; an increase was noticed in experimental group from (p < 0.001; p = 0.006 respectively). The mean value of psychological domain in the control group also decreased from 46.03 to 45.63 but was not statistically significant. It increased in the experimental group from 45.13 to 51.50 (p < 0.001). In the domain of social relationship the mean value in control group declined significantly (p = 0.032) and no improvement was seen in the experimental group. When all variables were compared between the groups it was found that physical and psychological domain showed statistically significant results in post intervention (p < 0.001; p < 0.001).

**Table 2:** Comparison of transformed scores of Physical, Psychological, social and environmental domain for within the group, and between the groups

**Variables**	**Group**	**Pre**	**Post**	**p-value**^**b**^	**% change**
		**Mean ± SD**	**Mean ± SD**		
**PHYS**	Control	39.19 ± 5.14	38.67 ± 4.98	**0.057**	−1.32%
	Experimental	37.30 ± 5.42	45.43 ± 7.32	**<0.0001**	+21.79%
	**p-value**^**a**^	**0.120**	**<0.0001**		
**PSYCH**	Control	46.03 ± 3.08	45.63 ± 3.48	**0.160**	−0.40%
	Experimental	45.13 ± 3.52	51.50 ± 6.46	**<0.0001**	+14.09%
	**p-value**^**a**^	**0.237**	**<0.0001**		
**SOCIAL**	Control	29.67 ± 4.58	28.45 ± 5.26	**0.032 **	−1.21%
	Experimental	28.47 ± 7.00	28.24 ± 6.98	**0.800**	+0.81%
	**p-value**^**a**^	**0.370**	**0.879**		
**ENVIR**	Control	38.49 ± 1.77	38.18 ± 2.15	**0.044**	−0.30%
	Experimental	37.76 ± 2.28	38.62 ± 2.60	**0.006**	+2.98%
	**p-value**^**a**^	**0.119**	**0.417**		
**QOL**	Control	67.88 ± 3.14	67.39 ± 3.34	**0.011 **	
	Experimental	66.78 ± 3.68	71.36 ± 4.66	**<0.0001**	
	**p-value**^**a**^	**0.161**	**<0.0001**		

a-P value between groups.

b-P value within group.

## Discussion

In this open label randomized control trial, significant improvement was noted in the physical and psychological domains of WHOQOL-BREF after intervention with a novel occupational therapy program (OTP) in subjects with mild to moderate dementia.

In a systematic review of literature on association between cognitive impairment and QOL, a relationship though weak has been reported [29]. In addition to cognitive deficit, high prevalence of depression [30], loss of independence in activities of daily living [31] along with the behavioral and psychological symptoms have profoundly negative impact upon QOL [32]. Our study shows that the novel OTP improved physical performance, improving sleep quality and increased energy for activities of daily living. Alteration in psychological components was also noted such as a greater appreciation of life and a reduction in negative feeling such as anxiety, depression etc. This is also likely to be effective in preventing or delaying decline in QOL. It is possible that improved physical performance creates sense of independency, increased motivation, positive outlook and reduced behavioral and psychological symptoms. Recreational activities have the added advantage of creating a distraction from patient’s present illness.

A review of literature supports our results. In a similar study, Graff et al showed improvement in quality of life sustainable 12 weeks after occupational therapy intervention in a group of community living older patients with dementia and their caregivers [33]. Similar results were also noted by Dooley and Hinojosa [34]. Korczak et al demonstrated that occupational therapy provided at home could be cost effective in patients with dementia as it reduced the need for nursing care [35]. In a systematic review of seventeen studies Steultjens et al reported that occupational therapy interventions for community dwelling elderly people results in positive outcomes [36].

The novelty of the present study however lies in the breadth of the interventions. The OT program targeted the 5 domains namely; physical, functional, behavioral, psychological and cognitive; for intervention. No previous studies have tried to assess the simultaneous effect of therapy on these domains. Several intervention studies exist which have focused on solitary domains; namely; association between physical activity and dementia [37]; tailor made activities for improving psychosocial outcomes [38]; recreational interventions [39]; and physical activities [23]. A meta-analysis of studies including 751 cases of dementia reported small effect from sensory stimulation [40]. A list of twenty six potential occupational therapy interventions for dementia has been recently enumerated, most which of course focus on environmental modification, provision of assistive devices and referral to other sources [41]. The present study has been conducted in a hospital setting And the reproducibility in community needs to be examined.

### Limitation of our study

The study has a small sample size and a male predominance. There is an inherent observer bias in our study. Use of a generic QOL scale such as WHOQOL BREF was unavoidable as disease specific scales are not available in local language. A larger study with longer duration could probably address

## Conclusion

This open label randomized controlled trial employing a novel occupational therapy program improved quality of life of older patients with mild to moderate dementia in physical and psychological components of WHOQOL-BREF scale. Application of this program in clinical practice will require larger sample size and sustainability over longer duration and utility in community setting.

## Competing interests

All the authors have seen the final manuscript and approve it for submission. The authors have no competing interests in the publication of this manuscript to declare.

## Authors’ contributions

PK conceived, designed and obtained ethical approval apart from developing the intervention program and collecting data of the entire trial. He also analysed and prepared the first draft of the manuscript. SCT and RKT were also instrumental in conceiving the study. VG and AG were involved in Intellectual inputs, extensive restructuring and critical revisions of the manuscript. VS was involved in study planning, data analysis and interpretation. ABD initiated and conceived the study. He also revised the manuscript and guarantor. All authors read and approved the final manuscript.
